# Chondroitin Sulfate Disaccharides in the Gas Phase: Differentiation and Conformational Constraints

**DOI:** 10.1021/acs.jpca.1c02463

**Published:** 2021-05-12

**Authors:** Maike Lettow, Kim Greis, Márkó Grabarics, Jan Horlebein, Rebecca L. Miller, Gerard Meijer, Gert von Helden, Kevin Pagel

**Affiliations:** † Fritz-Haber-Institut der Max-Planck-Gesellschaft, Faradayweg 4-6, 14195 Berlin, Germany; ‡ Institut für Chemie und Biochemie, Freie Universität Berlin, Arnimallee 22, 14195 Berlin, Germany; § Copenhagen Center for Glycomics, Department of Cellular and Molecular Medicine, Faculty of Health Sciences, University of Copenhagen, Blegdamsvej 3, DK-2200 Copenhagen N, Denmark

## Abstract

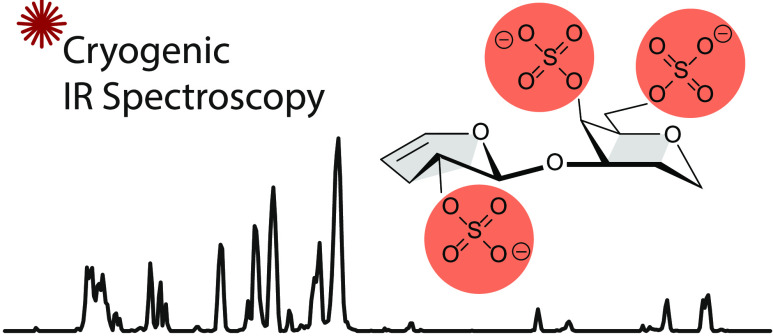

Glycosaminoglycans (GAGs) are a family of complex carbohydrates vital to all mammalian organisms and involved in numerous biological processes. Chondroitin and dermatan sulfate, an important class of GAGs, are linear macromolecules consisting of disaccharide building blocks of *N*-acetylgalactosamine and two different uronic acids. The varying degree and the site of sulfation render their characterization challenging. Here, we combine mass spectrometry with cryogenic infrared spectroscopy in the wavenumber range from 1000 to 1800 cm^–1^. Fingerprint spectra were recorded for a comprehensive set of disaccharides bearing all known motifs of sulfation. In addition, state-of-the-art quantum chemical calculations were performed to aid the understanding of the differences in the experimental fingerprint spectra. The results show that the degree and position of charged sulfate groups define the size of the conformational landscape in the gas phase. The detailed understanding of cryogenic infrared spectroscopy for acidic and often highly sulfated glycans may pave the way to utilize the technique in fragment-based sequencing approaches.

## Introduction

Carbohydrates or glycans are biological macromolecules that make up a significant amount of organic matter on earth and fulfill essential functional and structural roles in living organisms.^1^ Glycosaminoglycans (GAGs) are a major family of glycans, which participate in biological processes, such as hemostasis, inflammation, cell migration, proliferation, and differentiation.^2−4^ GAGs are long, acidic, and often sulfated, and within this family, an important class is chondroitin sulfate and dermatan sulfate (CS/DS). CS/DS are usually a part of larger glycoproteins, so-called proteoglycans, and often have chain lengths of over 50 disaccharide units.^5−7^ A characteristic for the glycan backbone of CS/DS are repeating disaccharide units of two different uronic acids (UA) linked to *N*-acetylgalactosamine (GalNAc). The UA in CS is glucuronic acid (GlcA), whereas the backbone of DS contains iduronic acid (IdoA) monosaccharides. Disaccharide fragments from larger chains are the product of bacterial chondroitinase digestion, which introduces a double bond between C4 and C5 in the UA (then abbreviated ΔUA). Varying O-sulfations at C2 of the uronic acid and at C4 and C6 of the GalNAc further increase the complexity of CS/DS chains significantly.^8^ The most informative level of GAG analysis resides in its disaccharide building blocks; therefore, disaccharide analysis is essential for any oligosaccharide or full-length GAG sequence.

Recent developments in mass spectrometry-based techniques, such as electron-based dissociation^9−11^ and ion mobility-mass spectrometry,^12,13^ have improved the sensitivity and integrity in GAG analysis toward varying uronic acid stereochemistry and in sulfation.^14,15^ Nevertheless, the distinction of certain sulfation motifs, especially O-sulfations at C4 and C6 in CS/DS, is still exceptionally challenging by mass spectrometry-based techniques.^13,16−20^

A novel approach for GAG structural characterization is infrared (IR) spectroscopy of ions in the gas phase.^21^ GAGs and their fragments ranging from monosaccharides to pentasaccharides have shown to exhibit a wealth of characteristic spectral features.^22−25^ Cryogenic temperatures have enhanced the spectral quality significantly in the midinfrared^26^ and the OH-stretching region.^24^ Here, we assess the capability of cryogenic IR spectroscopy for the differentiation and structural characterization of uronic acids of CS/DS disaccharides with all known motifs of sulfation.

## Methods

### Sample Preparation

Glycosaminoglycans were purchased from Iduron (Alderley Edge, U.K.) and used without further purification. For each disaccharide, a stock solution of 1 mM in H_2_O was further diluted prior to use to yield a 50 μM analyte solution in H_2_O/MeOH (v/v, 1/1).

### Ion Mobility-Mass Spectrometry

Drift tube (DT) ion mobility-mass spectrometry measurements were performed on a modified Synapt G2-S HDMS instrument (Waters, Manchester, U.K.) containing a drift tube instead of the commercial traveling wave cell^27^ and equipped with a nanoelectrospray ionization source. Ion mobilities were determined employing the stepped-field approach, and collision cross sections (CCS) were derived from the respective mobilities using the Mason–Schamp equation.^28^

### Cryogenic Infrared Spectroscopy

The home-built experimental setup has been described in detail recently.^29−31^ In brief, anions are produced in a nanoelectrospray ionization source, mass-to-charge selected in a quadrupole mass filter, and stored in an ion trap at ca. 90 K. Generated in a pulsed Even-Lavie valve, superfluid helium nanodroplets traverse the ion trap and pick up single ions. It has been shown that in the event of the pick-up, the ions are, with the rate of cooling, kinetically trapped in their present conformation. In a detection region of the instrument, the doped nanodroplets overlap with the laser beam of the Fritz-Haber-Institut free-electron laser (FHI FEL). At a resonant wavelength, the ion is vibrationally excited and immediately cooled to its ground state by evaporative cooling of the helium bath. After several iterations, the ion is eventually released from the nanodroplet and detected in a time-of-flight mass analyzer. The ion signal is plotted as a function of the incident photon energy and divided by the corresponding laser power as a first-order approximation. Relative intensities are, therefore, considered as a general reference. Infrared spectra are the result of an average of two individual scans.

### Computational Methods

The conformational space of the disaccharides was sampled using the evolutionary algorithm FAFOOM^32^ utilizing external software FHI-aims^33^ for local DFT optimization of each sampled structure at the PBE + vdW^TS^^34^ level of theory and *light* basis set settings. Mutation of all rotatable bonds and ring puckers was allowed during the conformational search. The algorithm allows functional groups to interact. In total ca. 250 structures were sampled for each disaccharide using the described approach. The methodology has previously yielded excellent accuracy for small glycans.^35,36^

## Results and Discussion

**Figure 1:**
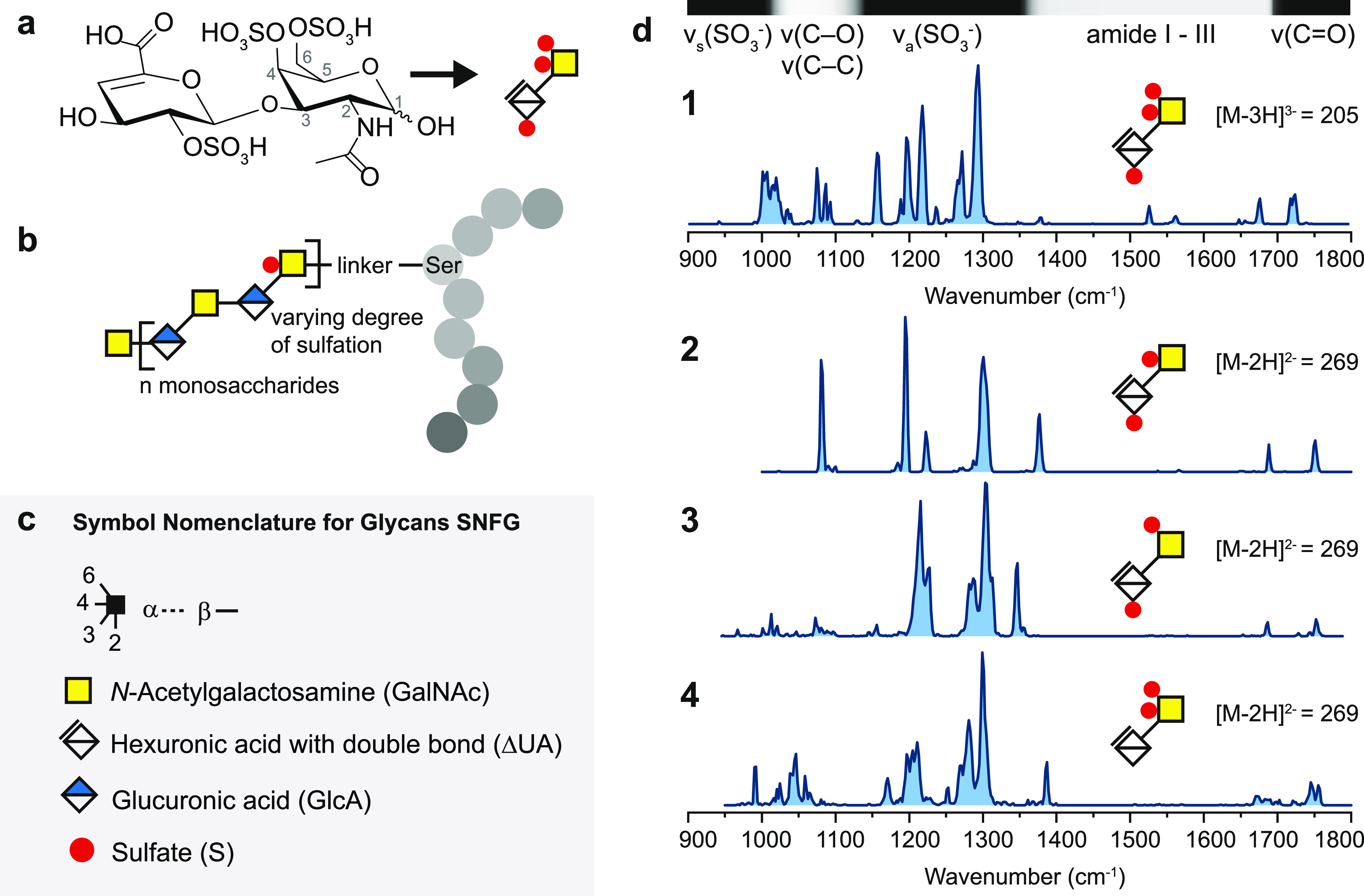
(a) Investigated chondroitin sulfate disaccharide **1** in chemical representation followed by its representation in the symbol nomenclature for glycans (SNFG).^37^ Both α and β anomers are present in the sample. (b) Representative structure of the proteoglycan bikunin, which carries one site for an O-linked chondroitin sulfate chain.^5,38−40^ The protein chain is depicted with gray circles and the three-letter code is used to highlight serine. (c) SNFG. (d) Cryogenic IR spectra of triply sulfated disaccharide **1** investigated as a [M – 3H]^3–^ anion with a mass-to-charge ratio (*m*/*z*) of 205 and doubly sulfated disaccharides **2**–4 investigated as [M – 2H]^2–^ isomeric anions with *m*/*z* of 269. On top of the first spectrum, the main vibrational features for these ions are qualitatively assigned in a horizontal bar.

Chondroitin sulfate disaccharides ranging from nonsulfated species up to triply sulfated species were chosen to cover all known sulfate combinations. In Figure 1a, the chemical structure of the triply sulfated disaccharide **1** is depicted. To assure molecular stability in the gas phase and limit charge migration, the disaccharides were investigated as deprotonated anionic species with a charge state equal to the number of sulfates at which the charges are localized.^23,26^ The nonsulfated disaccharide is investigated with a charge state equal to the number of carboxyl groups, i.e., as singly charged species.

**Figure 2:**
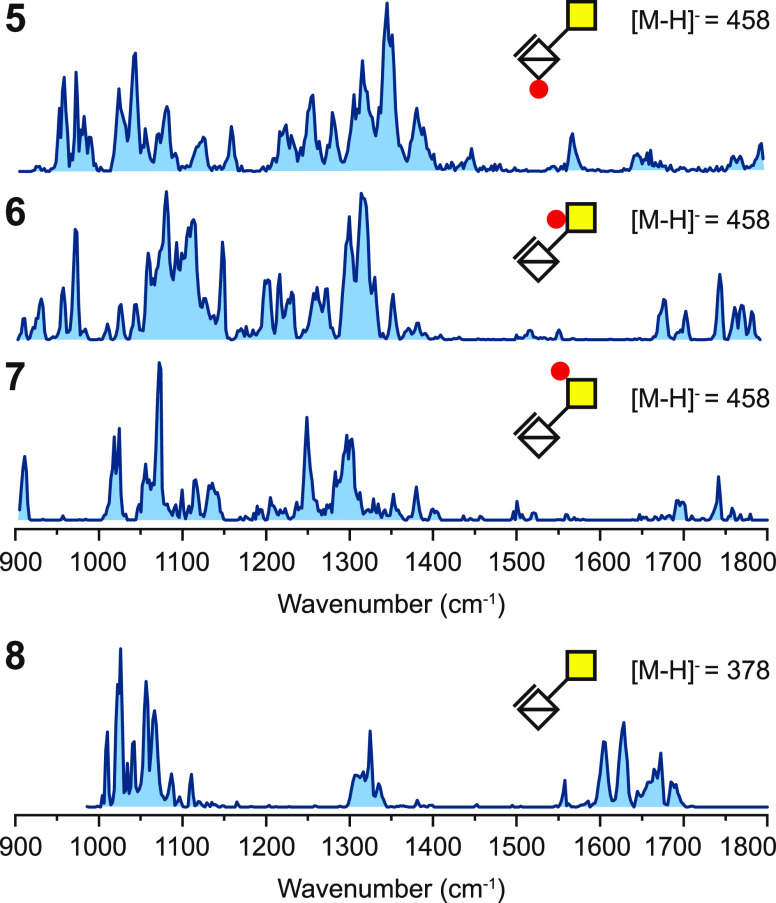
Cryogenic IR spectra of sulfated disaccharides **5**–7 investigated as [M – H]^−^ isomeric anions with *m*/*z* of 458 and nonsulfated disaccharide **8** investigated as a [M – H]^−^ anion with *m*/*z* of 378.

Cryogenic IR spectra in the mid-IR range were recorded at a minimum from 1000 to a maximum of 1800 cm^–1^, as shown in Figures 1d and 2. The assignment of spectral ranges to vibrational features is based on previous theoretical calculations of sulfated mono- and disaccharides.^23,25^ In brief, the spectral range below 1100 cm^–1^ is most characteristic for symmetric stretching of the charged sulfate ν_s_(SO_3_^–^) and overlaps the spectral range from 1000 to 1150 cm^–1^ in which ν(C–O) and ν(C–C) are found. From 1150 to 1350 cm^–1^, strong antisymmetric stretching vibrations of the charged sulfate ν_a_(SO_3_^–^) typically dominate the IR signature in sulfated glycosaminoglycans. Multiple minor vibrational features between 1200 and 1500 cm^–1^ correspond to C–H and O–H bending modes. Furthermore, above 1300 up to 1700 cm^–1^, the amide vibrations I to III are found in N-acetylated monosaccharides, of which amide I between 1600 and 1700 cm^–1^ is usually the most intense. Minor contributions of ν(C=C) in the range of amide I are characteristic for GAGs derived from lyase digestion. Above 1700 cm^–1^, ν(C=O) indicates the absence of a charged site at the carboxyl functional group. Carboxylate anions on the other hand yield an intense vibrational feature around 1650 cm^–1^, corresponding to the antisymmetric stretch ν_a_(COO^–^), whereas the potentially weaker ν_s_(COO^–^) is typically found between 1300 and 1400 cm^–1^.

The IR signature of the triply sulfated disaccharide **1** exhibits a unique absorption pattern with the strongest feature centered at 1292 cm^–1^ in the spectral range of ν_a_(SO_3_^–^). Single bands are, in some cases, close to the bandwidth of the free-electron laser (FWHM ca. 5 cm^–1^, i.e., 0.3–0.5% of the respective wavenumber). The IR signatures of the isomeric disaccharides **2** to **4** show a variety of well-resolved bands and are unambiguously distinguishable from each other. Especially, the IR signatures of disaccharides **2** and **3** with O-sulfation at C2 exhibit surprisingly narrow spectral features for ions of this size.

Although smaller in molecular size compared to the disaccharides with higher sulfation, the singly sulfated disaccharides **5** to **7** show a more complex IR signature, as shown in Figure 2. Especially the range up to 1400 cm^–1^ is more congested and shows less deviation between weak- and high-intensity signals. In disaccharide **6** carrying O-sulfation at *C*4, the range between 1650 and 1800 cm^–1^ shows a multitude of vibrational features for the two strongest potential oscillators in that range, i.e., C=O in the carboxyl and the amide groups. The nonsulfated disaccharide **8** was included to assess the impact of the backbone, and the IR spectrum clearly reveals that this ion carries the charge at the carboxylate.

The size of the ensemble of conformations present during the experiment correlates to the congestion of the IR spectrum and the number of vibrational features in a range in which a limited number of functional groups absorb. In our experimental setup, the ensemble of conformations is mainly defined by the temperature of the coolable ion trap (ca. 90 K). When the molecules are picked up by the superfluid helium nanodroplets (0.4 K), the rate of cooling is so fast that ions are kinetically trapped in local conformational minima.^41^ The IR spectra of the higher sulfated disaccharides are remarkably well resolved, whereas the IR spectra of the singly sulfated disaccharides are highly congested. Our hypothesis is that the higher sulfated and also higher charged disaccharides exhibit only a limited number of low-energy conformers, whilst the singly sulfated and also singly charged disaccharides exhibit a larger ensemble of low-energy conformers. To test this hypothesis, quantum chemical calculations were performed and the conformational space of the sulfated disaccharide β anomers was extensively sampled to yield ca. 250 conformers for each disaccharide. The β anomer was chosen under the premise that the stereocenter at C1 has only a minor influence on the overall conformation of the disaccharide. For each ion, all sampled conformers with relative energies (Δ*E*_PBE_) up to 50 kJ mol^–1^ were selected and characterized using two criteria: (1) the intramolecular distances of charged sulfates toward the hydrogens of the carboxyl and amide groups and (2) the dihedral angles Ψ(C1-O-C3-C4) and Φ(C2-C1-O-C3).

To qualitatively assess the conformational diversity, the diagrams relating relative energies to structural parameters and Ramachandran-type plots for glycosidic dihedral angles give insights into the orientation of selected functional groups and the glycan backbone, respectively. Key interactions in these molecules are hydrogen bonds of the charged sulfates toward the hydrogens of the carboxyl and amide groups. For this reason, the *x*-axis in the energy diagram shows the distances of all charged sulfates toward either the secondary amide of N-acetyl or the neutral carboxyl functional groups and on the *y*-axis of the relative energies. The different distances in a single conformation are, thus, given from left to right at the same relative energy. The Ramachandran-type plots for glycosidic dihedral angles, analogous to Ramachandran plots of peptides and proteins, show the dihedral angles Ψ(C1-O-C3-C4) and Φ(C2-C1-O-C3) on *y*- and *x*-axes, respectively, with dots for single conformers, which are colored according to the relative energy.

**Figure 3:**
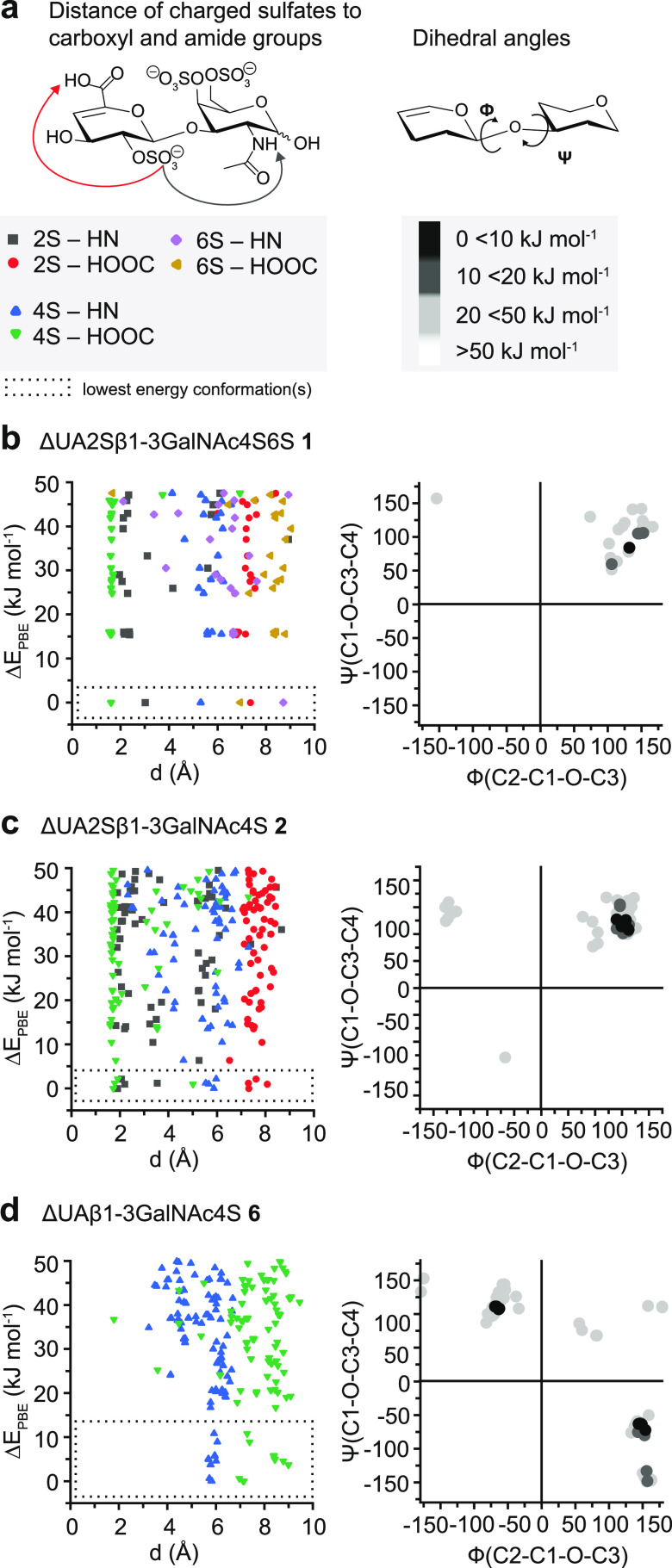
(a) Left panel: calculation of the intramolecular distance of selected functional groups in Å in conformers with relative energies Δ*E*_PBE_ < 50 kJ mol^–1^. Right panel: calculation of the dihedral angles Ψ(C1-O-C3-C4) and Φ(C2-C1-O-C3) in the degree at the glycosidic bond with respect to the relative energies of the conformers. The results are presented in Ramachandran-type plots for glycosidic linkages. (b–d) Results for the triply sulfated disaccharide **1**, doubly sulfated disaccharide **2**, and singly sulfated disaccharide **6**. Generally, an increase in conformational heterogeneity is observed with a decreasing extent of sulfation and charge.

The conformers of disaccharide **1**, Figure 3b, with three charged sulfate groups show trends in the orientation of the sulfate groups. More importantly, one conformer is with 15 kJ mol^–1^ significantly lower in energy than the second- and third-lowest-energy conformers. The hydrogen-bonding network is dominated by the interaction between the O-sulfate group at C4 and the neutral carboxyl functional group but also by the O-sulfate group at C2 and the secondary amide. The O-sulfate group at C6 has in all conformers a distance larger than 5 Å toward the amide and carboxyl to minimize Coulombic repulsion with the sulfate groups in their proximity. The Ramachandran-type plot shows a very narrow distribution, indicating similarities in the glycan backbone of the low-energy conformers. Conformers of high similarity are more likely to relax into the same local minimum or the global minimum in the cold ion trap. Disaccharide **2**, Figure 3c, with two charged sulfate groups likewise reveals trends in the energy diagram, yet, far more conformers are within 20 kJ mol^–1^ of the lowest-energy conformer. The distribution in the Ramachandran-type plot is narrow. In contrast, O-sulfate at C4 does not form a hydrogen bond with the neutral carboxyl in the singly sulfated disaccharide **6**, Figure 3d. The Ramachandran-type plot shows that two clusters of conformers with large differences in their glycan backbone are present. This can indicate that at least two but probably several local minima are populated that are conformationally constrained to reach the same or global minimum structure. The plots of the disaccharides **3**–**5** and **7**, in Figure S1, further support the observed trend of a growing conformational heterogeneity in chondroitin sulfate ions with a decreasing degree of charged groups, which are sulfate groups in this case.

For the discussed disaccharides **1**, **2**, and **6**, selected low-energy conformers were reoptimized at a higher level of theory. Theoretical infrared spectra were computed for the lowest-energy conformers and compared to the experimental spectra, Figure S2. The match between computed and experimental spectra is satisfying, yet, anharmonicities challenge the calculations for glycosaminoglycans and extend the computational effort.^42^

In addition to gas-phase IR spectroscopy, drift tube ion mobility-mass spectrometry (DTIM-MS) experiments were performed to compare the potential of the method for disaccharide differentiation. The rotationally averaged collision cross section (CCS) reflects the shape of an ion and is an instrument-independent physical property. CCS values of the disaccharides, measured in nitrogen and helium, are summarized in Table 1. The most challenging isomeric disaccharides **6** and **7** with O-sulfate either at C4 or at C6 are not distinguishable based on their CCS. The results indicate that differentiating certain isomeric disaccharides in sets **2–4** and **5–7** is possible but may require IMS instrumentation with resolving power on the order of 100 when they are present in a mixture and measurement uncertainties below 0.5% when present alone.

**Table 1:** Collision Cross Sections (CCSs) of the Investigated Chondroitin Sulfate Disaccharides

	molecule	z	*m*/*z*^a^	^DT^CCS_N2_^b^	^DT^CCS _He_^b^
**1**	ΔUA2Sβ1-3GalNAc4S6S	–3	205	317	160
**2**	ΔUA2Sβ1-3GalNAc4S	–2	269	241	138
**3**	ΔUA2Sβ1-3GalNAc6S	–2	269	244	140
**4**	ΔUAβ1-3GalNAc4S6S	–2	269	239	135
**5**	ΔUA2Sβ1-3GalNAc	–1	458	190	127
**6**	ΔUAβ1-3GalNAc4S	–1	458	189	122
**7**	ΔUAβ1-3GalNAc6S	–1	458	189	122
**8**	ΔUAβ1-3GalNAc	–1	378	178	112

a Monoisotopic mass-to-charge (*m*/*z*) ratio.

b In Å^2^, DT: drift tube.

## Conclusions

In summary, we show here that cryogenic IR spectroscopy has the potential to unambiguously differentiate all known sulfation patterns in uronic acids of chondroitin sulfate and dermatan sulfate disaccharides, including the distinction of 4O- and 6O-sulfation, a long-standing challenge in GAG analysis. Identification of disaccharide isomers based purely on their ion mobility-derived CCS values is partially ambiguous and requires outstanding resolving power. Furthermore, we show that for chondroitin sulfate ions with a low charge state and a low degree of sulfation, large ensembles of low-energy conformers are present. Whereas for higher charged and higher sulfated chondroitin sulfate ions, only a few low-energy conformers are present. This conformational constraint in highly charged gas-phase ions has also been described for other biomolecules such as peptides and proteins.^43^ Yet, additional experiments are necessary to evaluate whether this effect purely results from Coulombic repulsion or also from the nature of the O-sulfate groups, which, in contrast to more localized carboxylates, potentially induce further constraints with their steric repulsion^44^ and conformational flexibility.

## Supporting Information Available

Experimental details on computational methods and coordinates of lowest-energy conformers; the energy diagram and Ramachandran-type plots for disaccharides 3, 4, 5, and 7; and the comparison of experimental and computed infrared spectra for disaccharides 1, 2, and 6 (PDF)
