# Supplementary material for: Chondroitin Sulfate Disaccharides in the Gas Phase: Differentiation and Conformational Constraints
Source: J Phys Chem A. 2021 May 12;125(20):4373–9. doi: 10.1021/acs.jpca.1c02463 (PMC8279649; doi:10.1021/acs.jpca.1c02463)
Supplement: Supplementary file 1 — jp1c02463_si_001.pdf [file jp1c02463_si_001.pdf]

Chondroitin Sulfate Disaccharides in the Gas Phase: Differentiation and Conformational Constraints

Maike Lettow, Kim Greis, Márkó Grabarics, Jan Horlebein, Rebecca L. Miller, Gerard Meijer, Gert von Helden and Kevin Pagel**

Maike Lettow, Kim Greis, Márkó Grabarics, Jan Horlebein, Kevin Pagel – Fritz-Haber-Institut der Max-Planck-Gesellschaft, Faradayweg 4–6, 14195 Berlin, Germany; Institut für Chemie und Biochemie, Freie Universität Berlin, Arnimallee 22, 14195 Berlin, Germany;

Gerard Meijer, Gert von Helden – Fritz-Haber-Institut der Max-Planck-Gesellschaft, Faradayweg 4–6, 14195 Berlin, Germany;

Rebecca L. Miller – Copenhagen Center for Glycomics, Department of Cellular and Molecular Medicine, Faculty of Health Sciences, University of Copenhagen, Blegdamsvej 3, DK-2200 Copenhagen N, Denmark;

*Corresponding authors: maikelettow@fhi-berlin.mpg.de, kevin.pagel@fu-berlin.de

Table of Contents

Experimental Details S1

Computational Methods S1

Figures S3

Figure S2. Energy diagram and Ramachandran-type plots for glycosidic linkages for disaccharides **3**, **4**, **5** and **7** in (a) – (d). S3

Figure S2. Experimental and computed infrared spectra of disaccharides **1**, **2** and **6** in (a) – (c). S4

Tables S5

Table S1. FAFOOM settings used for sampling the conformational space of the disaccharides. S5

Table S2. Sampled and reoptimized structures. S6

Table S3. Relative energetics of a subset of sampled structures in kJ mol^{-1} for ΔUA2Sβ1-3GalNAc4S6S. ... S7

Table S4. Relative energetics of a subset of sampled structures in kJ mol^{-1} for ΔUA2Sβ1-3GalNAc4S. S8

Table S5. Relative energetics of a subset of sampled structures in kJ mol^{-1} for ΔUAβ1-3GalNAc4S. S9

Coordinates of lowest-energy conformers S10

References S13

Experimental Details

Computational Methods

The conformational space of the disaccharides was sampled using the evolutionary algorithm FAFOOM[1] utilizing the external software FHI-aims[2] for local DFT optimization of each sampled structure at the PBE+vdW^{TS}[3, 4] level of theory using *light* basis set settings. Mutation of all rotatable bonds and ring puckers was allowed during the conformational search. The algorithm allows functional groups to interact. The parameters of the genetic algorithm are specified in **Table S1**. In total 10 algorithm runs were run separately for each disaccharide. The methodology has previously yielded excellent accuracy for small glycans[5, 6]. Bond distances, dihedral angles and ring puckers were extracted from the sampled structures using a self-written python script.

To assess the quality of the sampled structures and their energetics, a subset of structures with a single-point energy (ΔE_{PBE}) below ca. 20 kJ mol^{-1} (and selected structures above the threshold carrying distinct motifs) were reoptimized at the hybrid DFT level PBE0+D3[7, 8] in Gaussian 16[9] for the disaccharides ΔUA2Sβ1-3GalNAc4S6S, ΔUA2Sβ1-3GalNAc4S and ΔUAβ1-3GalNAc4S. The basis set 6-31G(d) was chosen for all atoms except sulfur, for which the basis set 6-311+G(2df,2dp) was used. This method has previously been used for theoretically modelling of glycosaminoglycans[10]. For sulfur in higher oxidation states, a small basis set fails to optimize into a reasonable structure. Using the mixed basis set approach is a reasonable compromise between computational cost and chemical accuracy. The number of sampled and reoptimized structures is summarized in **Table S2**. A smaller subset of structures was chosen for reoptimization and frequency calculation at the PBE0+D3/6-311+G(2df,2dp) level of theory.

Energetics

The relative energetics of initial sampling ΔE_{PBE}, reoptimization with mixed basis sets $\Delta E_{PBE0,mixed}$ and reoptimization with only one basis set ΔE_{PBE0} are shown in **Table S3** for ΔUA2Sβ1-3GalNAc4S6S, **Table S4** for ΔUA2Sβ1-3GalNAc4S and **Table S5** for ΔUAβ1-3GalNAc4S. Furthermore, the free energy at 90 K, $\Delta F_{PBE0,90K}$, similar to the experimental conditions at which the spectra were recorded, and the ring puckers P of the sampled structures are shown in the tables. The energetics of the sampled structures ΔE_{PBE} are generally slightly underestimated at PBE+vdW^{TS}/*light* level of theory, but are in the same order of magnitude and follow a similar hierarchy compared to the energetics ΔE_{PBE0} computed at the hybrid PBE0+D3/*mixed* level of theory in Gaussian 16.

Frequencies

Frequencies of selected structures were computed at the PBE0+D3/6-311+G(2df,2dp) level of theory and scaled by a factor of 0.965. Exemplary infrared spectra and their experimental homologues are shown in **Figure S2**. Generally, the carboxyl stretching and the amide I vibration are modelled well, with a slight shift in frequency, while the intensity of the amide II vibration is overemphasized in the theoretical spectrum. The lower-energy region of the theoretical spectrum, where commonly ν(C–O), ν(C–C), $\nu_a(SO_3^-)$ and $\nu_s(SO_3^-)$ can be found, is highly congested. The absorption bands in this region with good agreement with the experimental spectrum usually stem from $\nu_a(SO_3^-)$ and $\nu_s(SO_3^-)$ stretching modes. The absorption bands of the ν(C–O) and ν(C–C) are commonly more challenging to model in unprotected glycans due to anharmonicities, which are not accounted for in the harmonic approximation used for frequency calculation.

Figures

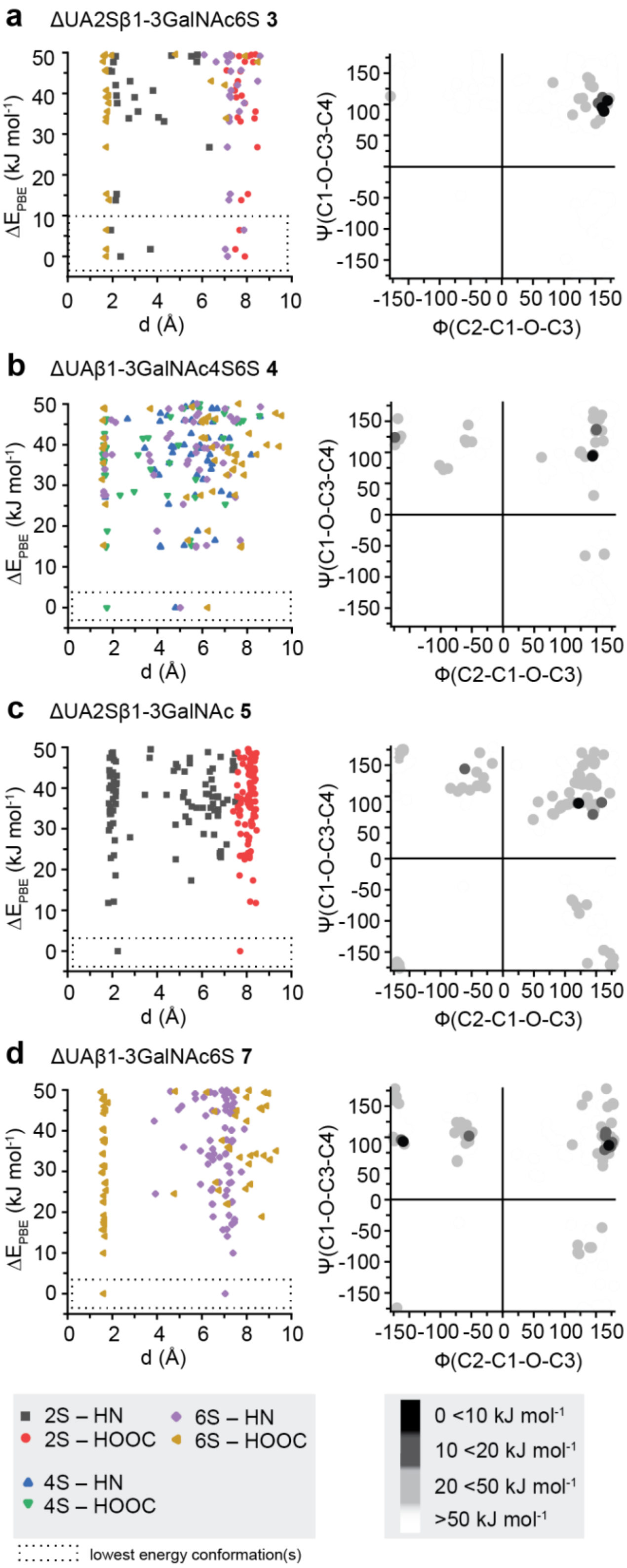

Figure S1. Energy diagram and Ramachandran-type plots for glycosidic linkages for disaccharides **3**, **4**, **5** and **7** in (a) – (d).

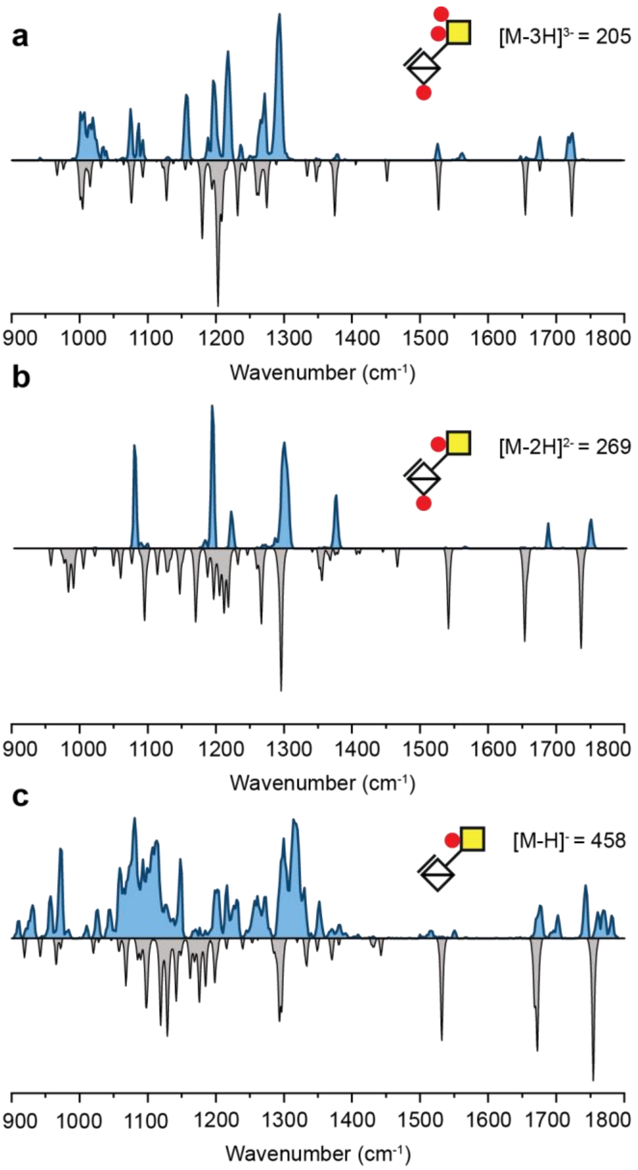

Figure S2. Experimental and computed infrared spectra of disaccharides **1**, **2** and **6** in (a) – (c). The experimental spectra are shown in the blue traces, while the computed spectra of the lowest-energy conformers are shown in the inverted gray traces.

Tables

Table S1. FAFOOM settings used for sampling the conformational space of the disaccharides.

	Parameter	Value
Molecule	Distance_cutoff_1	1.2
	Distance_cutoff_2	2.15
	Rmsd_cutoff_uniq	0.25
FAFOOM settings	Popsize	10
	Prob_for_crossing	0.95
	Prof_for_mut_pyranosering	0.6
	Prob_for_mut_torsion	0.8
	Fitness_sum_limit	1.2
	Selection	Roulette wheel
	Max_mutations_torsion	3
	Max_iter	30

Table S2. Sampled and reoptimized structures.

	**Molecule**	**# sampled structures**	**# reoptimized structures**
**1**	ΔUA2Sβ1-3GalNAc4S6S	217	12
**2**	ΔUA2Sβ1-3GalNAc4S	254	11
**3**	ΔUA2Sβ1-3GalNAc6S	267	-
**4**	ΔUAβ1-3GalNAc4S6S	212	-
**5**	ΔUA2Sβ1-3GalNAc	275	-
**6**	ΔUAβ1-3GalNAc4S	269	13
**7**	ΔUAβ1-3GalNAc6S	232	-
**8**	ΔUAβ1-3GalNAc	288	-

Table S3. Relative energetics of a subset of sampled structures in kJ mol^{-1} for ΔUA2Sβ1-3GalNAc4S6S.

ΔE_{PBE} is obtained from initial sampling, $\Delta E_{PBE0,mixed}$ from reoptimization with mixed basis sets, ΔE_{PBE0} and $\Delta F_{PBE0,90K}$ from reoptimization at PBE0+D3/6-311+G(2df,2dp) level of theory. ΔE_{PBE0} and $\Delta E_{PBE0,mixed}$ are including zero-point vibrational energy. The ring puckers $P_{\Delta UA}$ and P_{GalNAc} were derived from the sampled geometries.

**Conformer**	**$P_{\Delta UA}$**	**P_{GalNAc}**	**ΔE_{PBE}**	**$\Delta E_{PBE0,mixed}$**	**ΔE_{PBE0}**	**$\Delta F_{PBE0,90K}$**
conf_00	$^{2}H_{1}$	$^{4}C_{1}$	0.0	0.0	0.0	0.0
conf_01	E_{2}	$B_{O,3}$	15.4	28.4	-	-
conf_02	$^{2}H_{1}$	$B_{O,3}$	15.5	18.1	23.4	24.1
conf_03	$^{2}H_{3}$	$B_{O,3}$	16.0	19.0	25.9	27.8
conf_04	$^{1}H_{2}$	$^{1}S_{3}$	24.8	40.3	-	-
conf_05	$^{1}H_{2}$	$B_{O,3}$	26.0	39.7	40.6	40.7
conf_06	$^{2}H_{1}$	$^{2}S_{O}$	27.5	35.0	-	-
conf_07	$^{2}H_{1}$	$B_{O,3}$	27.9	40.7	-	-
conf_08	$^{2}H_{1}$	$^{O}S_{2}$	30.5	42.6	-	-
conf_09	$^{1}H_{2}$	$^{1}S_{5}$	45.7	59.6	-	-
conf_10	$^{1}H_{2}$	$^{4}C_{1}$	47.1	61.1	-	-
conf_11	^{1}E	$^{1}C_{4}$	47.5	54.2	-	-

Table S4. Relative energetics of a subset of sampled structures in kJ mol^{-1} for ΔUA2Sβ1-3GalNAc4S.

ΔE_{PBE} is obtained from initial sampling, $\Delta E_{PBE0,mixed}$ from reoptimization with mixed basis sets, ΔE_{PBE0} and $\Delta F_{PBE0,90K}$ from reoptimization at PBE0+D3/6-311+G(2df,2dp) level of theory. ΔE_{PBE0} and $\Delta E_{PBE0,mixed}$ are including zero-point vibrational energy. The ring puckers $P_{\Delta UA}$ and P_{GalNAc} were derived from the sampled geometries.

**Conformer**	$P_{\Delta UA}$	P_{GalNAc}	ΔE_{PBE}	$\Delta E_{PBE0,mixed}$	ΔE_{PBE0}	$\Delta F_{PBE0,90K}$
conf_00	$^{2}S_{O}$	$^{4}C_{1}$	0.0	0.0	0.0	0.0
conf_01	$^{1}H_{2}$	$^{4}C_{1}$	1.0	14.3	5.9	7.0
conf_02	$B_{O,3}$	$^{1}S_{3}$	2.1	11.8	-	-
conf_03	$^{2}H_{1}$	$^{O}S_{2}$	6.4	16.1	9.2	10.9
conf_04	$B_{O,3}$	$^{4}C_{1}$	10.5	21.1	17.5	20.1
conf_05	$^{1}H_{2}$	$^{4}C_{1}$	13.5	27.6	-	-
conf_06	$B_{O,3}$	$^{4}C_{1}$	14.3	23.3	-	-
conf_07	$^{2}S_{O}$	$^{4}C_{1}$	14.6	26.0	-	-
conf_08	$^{2}S_{O}$	$^{4}C_{1}$	15.7	24.5	-	-
conf_09	$B_{O,3}$	$^{O,3}B$	18.1	29.7	-	-
conf_10	$B_{O,3}$	$^{O,3}B$	40.9	52.6	45.2	44.8

Table S5. Relative energetics of a subset of sampled structures in kJ mol^{-1} for ΔUAβ1-3GalNAc4S.

ΔE_{PBE} is obtained from initial sampling, $\Delta E_{PBE0,mixed}$ from reoptimization with mixed basis sets, ΔE_{PBE0} and $\Delta F_{PBE0,90K}$ from reoptimization at PBE0+D3/6-311+G(2df,2dp) level of theory. ΔE_{PBE0} and $\Delta E_{PBE0,mixed}$ are including zero-point vibrational energy. The ring puckers $P_{\Delta UA}$ and P_{GalNAc} were derived from the sampled geometries.

**Conformer**	$P_{\Delta UA}$	P_{GalNAc}	ΔE_{PBE}	$\Delta E_{PBE0,mixed}$	ΔE_{PBE0}	$\Delta F_{PBE0,90K}$
conf_00	$^{2}H_{1}$	$^{4}C_{1}$	0.0	1.8	1.2	2.2
conf_01	$^{2}H_{1}$	$^{4}C_{1}$	0.6	0.0	1.5	2.1
conf_02	$^{2}H_{3}$	$^{4}C_{1}$	3.7	1.6	0.0	0.0
conf_03	$^{2}H_{3}$	$^{4}C_{1}$	4.6	1.1	1.9	1.8
conf_04	$^{2}H_{3}$	$^{4}C_{1}$	4.9	2.6	-	-
conf_05	$^{2}H_{3}$	$^{4}C_{1}$	5.7	2.1	-	-
conf_06	$^{2}H_{1}$	$^{4}C_{1}$	8.9	8.0	-	-
conf_07	$^{2}H_{1}$	$^{1}S_{3}$	10.8	13.0	-	-
conf_08	^{2}E	$^{4}C_{1}$	16.7	12.7	-	-
conf_09	$^{2}H_{1}$	$^{4}C_{1}$	17.9	22.8	-	-
conf_10	$^{2}H_{3}$	$^{1,4}B$	22.5	24.8	-	-
conf_11	$^{2}S_{O}$	$^{1}C_{4}$	24.0	19.9	29.7	29.8
conf_12	$B_{O,3}$	$^{4}C_{1}$	36.7	33.2	34.3	35.8

Coordinates of lowest-energy conformers

The coordinates of the structures optimized at PBE0+D3/6-311+G(2df,2dp) level of are given.

ΔUA2Sβ1-3GalNAc4S6S/conf_00

Charge = -3, Multiplicity = 1

C	-3.63793100	1.41577800	-1.63058700
C	-3.03498500	0.44406400	-0.61680200
C	-1.60391900	0.86484000	-0.29783200
C	-2.30866500	3.08930100	-0.37374300
C	-3.24842800	2.81305500	-1.27607300
H	-3.61989800	0.44261600	0.30727200
H	-3.20410000	1.14999600	-2.60930700
H	-3.77016500	3.63613900	-1.74282200
H	-1.01893900	0.90904300	-1.22900700
O	-1.59439700	2.15545500	0.28453900
O	-2.93839200	-0.87458900	-1.13476100
O	-5.04115000	1.35072500	-1.70627200
H	-5.26707800	0.41262500	-1.86655200
C	-1.94687800	4.49930100	-0.00593100
O	-2.67233700	5.42491000	-0.30783300
C	1.41882700	0.25513100	0.35043000
C	0.17207300	-0.62000400	0.22392000
C	2.61863500	-0.62861000	0.00140300
C	0.29912500	-1.86662200	1.07900900
H	0.09449900	-0.92948100	-0.82799200
H	2.47344800	-0.99270200	-1.02638400
C	1.59899600	-2.59835800	0.71521200
H	0.37667000	-1.56547100	2.12817200
O	2.67875900	-1.72964100	0.89106000
O	-1.03394600	0.01088000	0.60803500
N	-0.88320100	-2.68761800	0.90227500
H	-1.55566300	-2.39215300	0.20761800
C	-1.29633600	-3.56539900	1.82817800
C	3.94901100	0.09117200	0.01951700
H	3.86899500	1.03152600	-0.53110600
H	4.22498000	0.33264700	1.04548800
O	-0.59345700	-3.90161900	2.78540400
C	-2.68307300	-4.10757800	1.63725100
H	-3.40397300	-3.38922800	2.03409300
H	-2.93338800	-4.23007100	0.58335600
H	-2.77012000	-5.04861300	2.17705400
H	1.55877200	-2.89969900	-0.34736800
O	1.80305200	-3.72169200	1.49361300
H	1.08925800	-3.77532000	2.15426500
H	1.35378500	1.08478800	-0.35865100
O	-0.83184600	4.67724800	0.65112900
H	-0.20819600	3.88427500	0.75052400
S	-4.24950500	-1.85970600	-1.03217100
O	-5.20044900	-1.33586100	-2.01650700
O	-3.66213800	-3.13103900	-1.39779600
O	-4.71296600	-1.74849400	0.33751200
O	1.46576700	0.75749300	1.67023000
S	1.71467300	2.35168900	1.95186900
O	1.00248000	2.55854000	3.19093000
O	3.14950600	2.52321900	2.01327400
O	1.11764400	3.03478500	0.78909100
O	4.98403700	-0.71658600	-0.51947100
S	5.31221000	-0.51948200	-2.11561800
O	5.76308700	0.85942800	-2.25300000
O	6.34652300	-1.51929400	-2.31671000
O	4.05482100	-0.78937400	-2.80666200

ΔUA2Sβ1-3GalNAc4S/conf_00

Charge = -2, Multiplicity = 1

C	2.44442100	-2.81976200	-0.21487400
C	2.02322200	-1.49185100	0.43179700
C	1.01555900	-0.83317200	-0.49662500
C	0.09598400	-3.02406300	-0.64284300
C	1.19335600	-3.63054100	-0.19332600
H	1.51746900	-1.71774700	1.37240900
H	2.72473000	-2.61464400	-1.25777800
H	1.14987000	-4.60560900	0.26894900
H	1.53347100	-0.29741500	-1.29457900
O	0.19199100	-1.77473800	-1.17757400
O	3.06445700	-0.61535100	0.82381400
O	3.50565600	-3.44449700	0.44906400
H	4.26400100	-2.87079300	0.26825000
C	-1.27893600	-3.58853600	-0.56250000
O	-1.52302500	-4.64909100	-0.04359700
C	-2.00770900	0.58190200	-0.45553300
C	-0.52521100	0.93256600	-0.48518300
C	-2.77521100	1.66397600	-1.21180700
C	-0.27769200	2.34825200	0.00697400
H	-0.21963100	0.88618200	-1.54118300
H	-2.48340800	1.58979600	-2.27293200
C	-1.11927300	3.29924800	-0.85211500
H	-0.62849600	2.43999300	1.03968900
O	-2.47716300	2.95314500	-0.72077800
O	0.25624300	0.03781100	0.26179900
N	1.14234500	2.63579800	-0.04537300
H	1.77671600	1.93009600	-0.40942800
C	1.69482300	3.61025300	0.69292900
C	-4.28356400	1.50397600	-1.10696300
H	-4.56365600	1.62629400	-0.05534800
H	-4.74628900	2.31504500	-1.67531800
O	1.02000500	4.47094900	1.26639200
O	-4.73747200	0.28681100	-1.63100900
C	3.19046100	3.58260700	0.79854500
H	3.65847300	3.03590100	-0.01823000
H	3.56151600	4.60449800	0.85681300
H	3.46283400	3.06064900	1.71834500
H	-4.53493100	-0.39935600	-0.96763700
H	-0.83235000	3.16841900	-1.91355700
O	-0.97936000	4.62198600	-0.50696900
H	-0.38946100	4.67796600	0.27343400
H	-2.13852900	-0.37736600	-0.95816000
O	-2.17426400	-2.79181400	-1.13974000
H	-2.96474800	-2.63247200	-0.57152300
S	4.16969200	-0.02994400	-0.22660600
O	3.41912000	0.82690300	-1.14737600
O	5.05671300	0.68511700	0.65892900
O	4.74444400	-1.20816000	-0.86858100
O	-2.48990900	0.51555600	0.87599700
S	-3.06336100	-0.90785100	1.44332700
O	-1.92192400	-1.75410800	1.69579200
O	-3.82728800	-0.47673500	2.58470000
O	-3.90155800	-1.44308800	0.33626000

ΔUAβ1-3GalNAc4S/conf_02

Charge = -1, Multiplicity = 1

C	-1.72599500	-3.43371400	-0.04177300
C	-0.58503400	-2.45049800	-0.23659300
C	-1.05095000	-1.26710000	-1.08036800
C	-3.10011700	-1.44346900	0.19798000
C	-2.90237700	-2.72220900	0.51702700
H	-0.29671400	-2.09083800	0.76329200
H	-1.98272100	-3.83236700	-1.03779400
H	-3.58063700	-3.22998000	1.18599800
H	-1.19618600	-1.60108300	-2.11027500
O	-2.30713200	-0.71810700	-0.61596000
O	-1.33899800	-4.48684500	0.80377500
H	-0.45197000	-4.73142200	0.51668600
C	-4.21991600	-0.62463100	0.70569300
O	-4.35016400	0.55190600	0.48201300
C	1.57248000	0.16604000	0.51187900
C	0.14621000	0.44512400	0.05752400
C	1.88126400	1.04345800	1.72393700
C	-0.08295000	1.93539600	-0.13752900
H	-0.52525100	0.10784800	0.85732300
H	1.21485800	0.71572900	2.54041600
C	0.31096200	2.67344500	1.14749900
H	0.56801000	2.29511700	-0.93992100
O	1.65708500	2.40345900	1.43942900
O	-0.16767800	-0.22066000	-1.15403200
N	-1.46410300	2.16702200	-0.49715200
H	-2.10941400	1.39318800	-0.42633800
C	-1.86157300	3.26729900	-1.15429800
C	3.31695400	0.90777800	2.20211400
H	3.98257000	1.23772000	1.39819900
H	3.45041000	1.58353900	3.05019300
O	-1.12195700	4.22894400	-1.34937300
O	3.60591400	-0.39704400	2.61842100
C	-3.28000500	3.25315400	-1.65383600
H	-3.86898600	2.44460500	-1.22305800
H	-3.74079300	4.21345700	-1.42650100
H	-3.25888800	3.14408300	-2.73989300
H	3.82045700	-0.89786500	1.81038800
H	-0.30656800	2.29128700	1.98302700
O	0.14259800	4.03299300	1.06030100
H	-0.08492900	4.26947000	0.14303300
H	1.65698800	-0.87918500	0.82189600
O	-5.08978400	-1.31683800	1.45841200
H	-5.75029900	-0.67907900	1.75291300
O	2.47585500	0.43829600	-0.53715900
S	3.37754200	-0.82360100	-1.07580700
O	2.42374200	-1.73494900	-1.70951500
O	4.28533200	-0.17929700	-1.97999300
O	3.96829800	-1.40420700	0.12721900
O	0.43469200	-3.15584100	-0.85455500
H	1.21199100	-2.58067400	-1.09401100

References

1. Supady, A.; Blum, V.; Baldauf, C., First-Principles Molecular Structure Search with a Genetic Algorithm. *J. Chem. Inf. Model.* **2015,** *55* (11), 2338-48.
2. Blum, V.; Gehrke, R.; Hanke, F.; Havu, P.; Havu, V.; Ren, X.; Reuter, K.; Scheffler, M., Ab initio molecular simulations with numeric atom-centered orbitals. *Comput. Phys. Commun.* **2009,** *180* (11), 2175-2196.
3. Tkatchenko, A.; Scheffler, M., Accurate molecular van der Waals interactions from ground-state electron density and free-atom reference data. *Phys. Rev. Lett.* **2009,** *102* (7), 073005.
4. Perdew, J. P.; Burke, K.; Ernzerhof, M., Generalized Gradient Approximation Made Simple. *Phys. Rev. Lett.* **1996,** *77* (18), 3865-3868.
5. Marianski, M.; Supady, A.; Ingram, T.; Schneider, M.; Baldauf, C., Assessing the Accuracy of Across-the-Scale Methods for Predicting Carbohydrate Conformational Energies for the Examples of Glucose and alpha-Maltose. *J. Chem. Theory Comput.* **2016,** *12* (12), 6157-6168.
6. Marianski, M.; Mucha, E.; Greis, K.; Moon, S.; Pardo, A.; Kirschbaum, C.; Thomas, D. A.; Meijer, G.; von Helden, G.; Gilmore, K., et al., Remote Participation during Glycosylation Reactions of Galactose Building Blocks: Direct Evidence from Cryogenic Vibrational Spectroscopy. *Angew. Chem. Int. Ed.* **2020,** *59* (15), 6166-6171.
7. Adamo, C.; Barone, V., Toward reliable density functional methods without adjustable parameters: The PBE0 model. *J. Chem. Phys.* **1999,** *110* (13), 6158-6170.
8. Grimme, S.; Antony, J.; Ehrlich, S.; Krieg, H., A consistent and accurate ab initio parametrization of density functional dispersion correction (DFT-D) for the 94 elements H-Pu. *J. Chem. Phys.* **2010,** *132* (15), 154104.
9. Frisch, M. J.; Trucks, G. W.; Schlegel, H. B.; Scuseria, G. E.; Robb, M. A.; Cheeseman, J. R.; Scalmani, G.; Barone, V.; Petersson, G. A.; Nakatsuji, H., et al. *Gaussian 16 Rev. A.03*, Wallingford, CT, 2016.
10. Lettow, M.; Grabarics, M.; Greis, K.; Mucha, E.; Thomas, D. A.; Chopra, P.; Boons, G. J.; Karlsson, R.; Turnbull, J. E.; Meijer, G., et al., Cryogenic Infrared Spectroscopy Reveals Structural Modularity in the Vibrational Fingerprints of Heparan Sulfate Diastereomers. *Anal. Chem.* **2020,** *92* (15), 10228-10232.
